# Enhanced Activity and Stability of an Acetyl Xylan Esterase in Hydrophilic Alcohols through Site-Directed Mutagenesis

**DOI:** 10.3390/molecules28217393

**Published:** 2023-11-02

**Authors:** Henry Madubuike, Natalie Ferry

**Affiliations:** School of Science Engineering and Environment, University of Salford, Manchester M5 4WT, UK

**Keywords:** protein engineering, biocatalyst, mutagenesis, acetyl xylan esterase, esterase

## Abstract

Current demands for the development of suitable biocatalysts showing high process performance is stimulated by the need to replace current chemical synthesis with cleaner alternatives. A drawback to the use of biocatalysts for unique applications is their low performance in industrial conditions. Hence, enzymes with improved performance are needed to achieve innovative and sustainable biocatalysis. In this study, we report the improved performance of an engineered acetyl xylan esterase (BaAXE) in a hydrophilic organic solvent. The structure of BaAXE was partitioned into a substrate-binding region and a solvent-affecting region. Using a rational design approach, charged residues were introduced at protein surfaces in the solvent-affecting region. Two sites present in the solvent-affecting region, A12D and Q143E, were selected for site-directed mutagenesis, which generated the mutants MUT12, MUT143 and MUT12-143. The mutants MUT12 and MUT143 reported lower Km (0.29 mM and 0.27 mM, respectively) compared to the wildtype (0.41 mM). The performance of the mutants in organic solvents was assessed after enzyme incubation in various strengths of alcohols. The mutants showed improved activity and stability compared to the wild type in low strengths of ethanol and methanol. However, the activity of MUT143 was lost in 40% methanol while MUT12 and MUT12-143 retained over 70% residual activity in this environment. Computational analysis links the improved performance of MUT12 and MUT12-143 to novel intermolecular interactions that are absent in MUT143. This work supports the rationale for protein engineering to augment the characteristics of wild-type proteins and provides more insight into the role of charged residues in conferring stability.

## 1. Introduction

Biocatalysts are fast replacing inorganic catalysts with enormous benefits in industrial properties due to their distinct properties [1]. Biocatalysts promotes clean reactions with low environmental damage, reactions with less toxic by-products, and the possibility of synthesising novel products that were not feasible using inorganic catalysts [2]. For example, biomass pretreatment using biocatalysts is a promising approach to achieving sustainable biorefining and would replace the need for harsh inorganic chemicals and energy-intense pretreatment techniques [3]. Despite the various advantages and undeniable potentials of enzyme-linked biocatalysis, the performance of enzymes in industrial conditions is still a major drawback to their applications. Enzymes have evolved to perform optimally in biological environments; however, in an industrial environment, the performance of an enzyme may be affected by the operating temperature, organic solvents in the reaction medium, inhibitory compounds, and substrate concentration [4,5]. Thus, the attempts to develop enzymes that overcome these limitations. The performance of an enzyme in industrial conditions can be enhanced using various approaches such as enzyme immobilization—cross-linked enzyme aggregate (CLEA), adsorption on an insoluble organic or inorganic support, entrapment in a carrier, encapsulation to membranes, and protein engineering—directed evolution and rational design [6,7,8]. Protein engineering can enhance various attributes of an enzyme such as thermostability, stability in organic solvent, substrate and product tolerance, stereoselectivity, activity, and introduction of new catalytic routes [2]. 

Directed evolution and rational design are the widely applied protein engineering strategies generating enzyme variants with modified catalytic or physiological properties [9,10,11]. In a rational design approach, the structure–function link of the target enzyme is exploited in formulating hypotheses, identifying hotspots for mutagenesis such as flexible regions, specific modification of the enzyme surface, or the modification of the catalytic site, thereby generating the desired genetic diversity [12,13,14]. For example, improved stability of a lipase from *Geobacillus stearothermophilus* in methanol was achieved by a rational design incorporating bulky aromatic residues to occupy solvent channels and induce aromatic interaction. In silico analysis was used to identify the tunnels and residues that were within 4–5 angstrom near the channel for mutagenesis study [15]. In contrast to directed evolution, which probes the molecular diversity generated by random mutagenesis to identify variants with improvements in the desired phenotypes, the rational design approach limits the size of the mutagenesis library and reduces screening and selection cycle [16]. The success of rational design strategies lies on adequate understanding of the structure and catalytic mechanism of the target enzyme hence, information on enzyme catalysis and the role of amino acid residues in the enzyme function would aid a smarter rational design [14]. For example, the catalytic mechanism of certain enzyme families such as the carboxylesterase superfamily (EC 3.3.1.1) has been largely understood and indispensable residues for catalysis have been established [17,18,19]. Using site-directed mutagenesis, the role of several residues in substrate binding and catalysis has been identified. Such mutagenesis studies helped in identifying residues required for catalysis and substrate binding and deciphering the mode of catalysis. For example, Glu28, Glu176 and Glu298 were identified as important residues for catalysis in α-l-arabinofuranosidase D3 from *Thermobacillus xylanilyticus* after site-directed mutagenesis of these residues to Ala, Asp or Gln resulted in weak or no activity [20]. Computational tools have aided in understanding, modelling, and developing novel variants [21]. The structure of target enzymes of known sequence can be modelled based on molecular mechanics resulting in 3D conformations with minimal potential energy. Modelling enzyme structures is beneficial for fast selection of mutant sites, especially for proteins with structures not yet developed and deposited in protein databases [21].

Enzymes are dynamic molecules exhibiting oscillatory motions and the dynamics of each part contribute to its tertiary structure, functional activity, and stability [22]. The functional characteristics of an enzyme are strongly linked to the folds forming the spatial structure, hence the three-dimensional structure is important in developing a mutagenesis strategy and identifying amino acid residues that would influence the desired properties [23]. For example, in rigidifying the protein structure (a strategy been explored for improving stability), the flexibility of amino acid residues or regions in a protein structure can be modulated [24,25,26]. Rigidifying the flexible regions is understood to enhance stability, while improving flexibility in regions is considered to enhance the activity of an enzyme [27,28]. In a study to enhance the activity and stability of lipase B from *Canadida antarctica* through site-directed mutagenesis exploring rational design, the enzyme structure was divided into two regions (substrate-binding regions and hydrophilic solvent-affecting region). Simultaneously modulating flexibility within these regions yielded variants with improved activity and stability [2]. However, the role of the introduced beneficial mutants has not been well characterised and is still debated in the literature over the role of certain amino acid groups in conferring stability. To target enhanced stability of enzymes in hydrophilic organic solvents, several studies and hypotheses have been developed. One of the strong propositions is the formation and strengthening of hydrated ion networks by charged amino acids [29]. Polar organic solvents have a devastating effect on the structure of enzymes because of their high degree of partitioning into the aqueous layer [30,31,32]. Loss of structural conformation caused by the disruption of the hydrogen bond network and hydrophobic interactions forming the water hydration shell of the protein resulting in the loss of enzyme activity in polar organic solvents [33,34,35]. A hydrated ion network prevents the aggregation of proteins via electrostatic charge repulsion and plays crucial roles in maintaining activity and improving resistance to hydrophilic organic solvents [34,36]. Hence, the stability of an enzyme is largely influenced by the intramolecular interactions of the functional groups and their interaction with the solvent environment [37]. Polar organic solvents tend to strip water off the enzyme surface, thereby dehydrating the enzyme so that activity is destroyed [38], thus considerations for improving stability targets water activity in and around the protein moiety are needed [39].

Esterases with novel properties are widely needed due to their potential in catalysing diverse reactions such as deacetylation of the xylan backbone, degradation of poly(ethylene terephthalate), and the synthesis of short-chain esters [40,41]. Biocatalysis in organic solvents is desired due to the possibility of performing reactions that are restricted kinetically or thermodynamically in an aqueous environment, improved solubility of substrates, easier product recovery, and because the possibility of side-reactions and inhibitory compounds occurrence is reduced [42]. Furthermore, some reactions perform better in polar organic solvents due to improved solubility of the substrates, hence biocatalysts that are stable in organic solvents are needed in industrial applications [41]. The stability of enzymes in a hydrophilic organic solvent is reduced due to the deforming effect of the penetrated solvents on the enzyme structure, which leads to a loss of activity [42]. Thus, strategies to rigidify the enzyme structure might develop beneficial mutants with improved stability in organic solvents. The biochemical and functional characterisation of a novel acetyl xylan esterase (BaAXE, EC 3.1.1.72) mined from the gut microbiota of the common black slug was previously reported [43,44,45]. BaAXE showed desirable characteristics of industrial enzymes and properties that can be utilized for biotechnological applications such as thermostability and moderate tolerance in polar and non-polar organic solvents [43]. Although BaAXE shows properties of a viable industrial enzyme, the enhancement of the enzyme properties has not been explored using protein engineering strategies. In this study, a rational design strategy was explored to develop beneficial mutants of BaAXE that expands the understanding of charged residues as suitable candidates for improving enzyme stability in organic solvents.

## 2. Results and Discussion

### 2.1. Enzyme Flexibility and Mutant Sites

**Figure 1:**
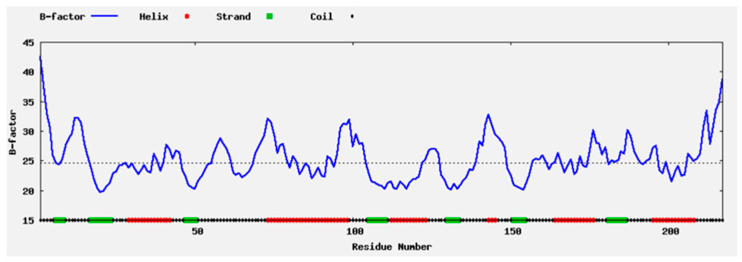
B-factor profile. The B-factor profile of BaAXE developed in ResQ shows the degree of flexibility in the different regions of the protein. The protein secondary structures are represented in different colours—helix in red, strand in green, and coil in black.

**Figure 2:**
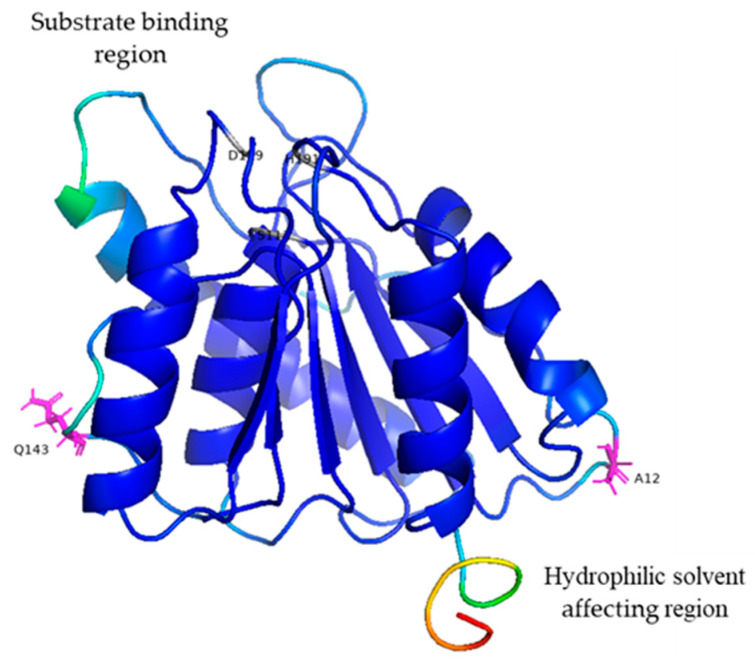
Orientation of BaAXE. The computationally developed structure of BaAXE was oriented to show the regions—substrate binding region, and the hydrophilic solvent affecting region. The active site residues (S111, H191 and D159) are shown in grey and mutant sites for stability enhancement are shown as sticks coloured in magenta. The protein model was annotated and displayed in PyMOL (https://pymol.org/2/).

The properties of the protein structure were computed in silico using the computationally solved structure of BaAXE. The highly flexible regions were mostly located within the loop structure while the sheets were less flexible (Figure 1), hence the loop region was selected for rigidifying the enzyme structure. The structure of BaAXE was partitioned into two regions—substrate binding and solvent affecting regions (Figure 2). Two residues located in the hydrophilic solvent affecting area were selected based on a strategy to introduce charged residues and intramolecular interactions, hence the development of the mutants MUT12, MUT143, and MUT12-143. The selected amino acid residues (A12 and Q143) were located on the loop structure near the N- and C-terminals, respectively, and reported high (A12) and intermediate (Q143) relative B-values (PROFbval) (Table 1) and A12 appears to be in proximity with D45 for a possible intermolecular interaction.

**Figure 3:**
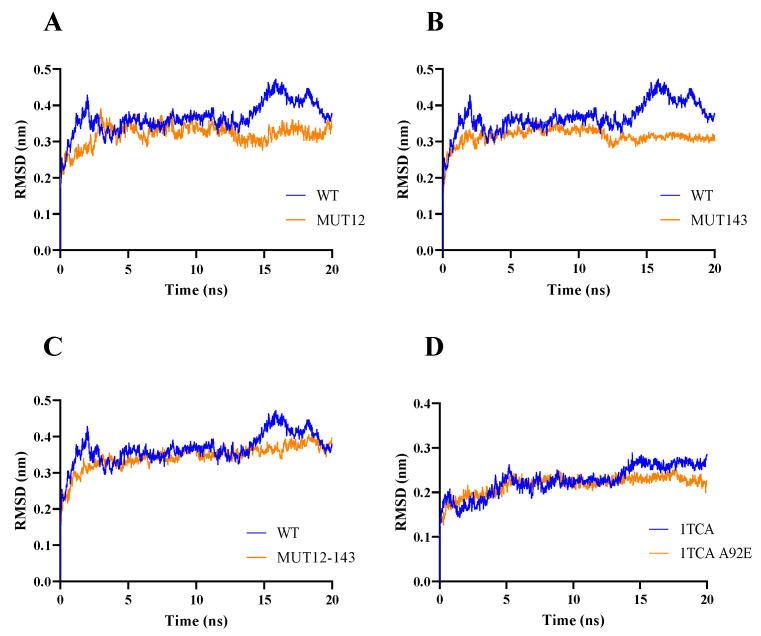
Molecular dynamics. The molecular dynamics of the WT and mutants was simulated using the computationally developed structures. Simulation data were generated for 20 ns for both WT (in blue) and mutants (in orange) with 1TCA simulations as reference. RMSD data were compared between BaAXE WT and (**A**) MUT12, (**B**) MUT143, (**C**) MUT12-143 and (**D**) 1TCA WT and 1TCA mutant (A92E). Molecular dynamics simulation was performed on WebGro and data presented in GraphPad Prism 9.

**Figure 4:**
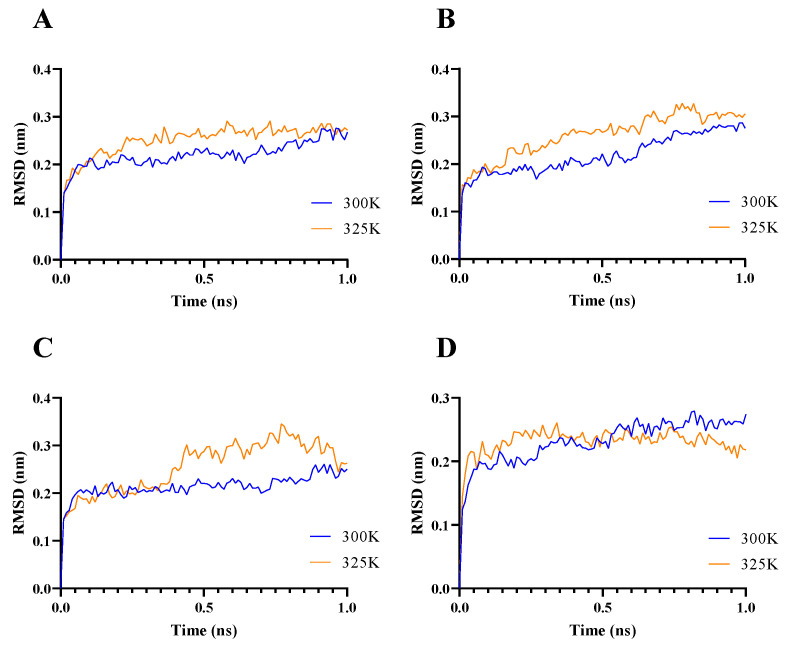
Flexibility analysis. The molecular dynamics of the WT and mutants was simulated using the computationally developed structures. Simulation data were generated for 1 ns for dynamics at temperature 300 K (in blue) and 325 K (in orange). RMSD data were compared between simulations in 300 K and 325 K. (**A**) WT, (**B**) MUT12, (**C**) MUT143, (**D**) MUT12-143. Molecular dynamics simulation was performed locally on a PC and data presented with GraphPad Prism 9.

**Figure 5:**
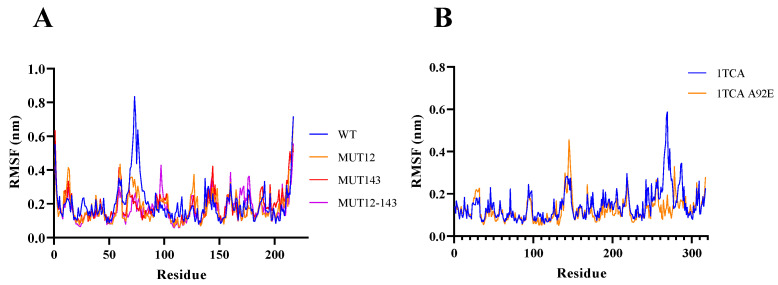
Flexibility per residue. The flexibility of each residue was simulated for WT and mutants. (**A**) The RMSF profile of WT and mutant—BaAXE. (**B**) The RMSF profile of WT and mutant—1TCA.

A flexibility analysis of BaAXE WT and mutants was performed using molecular dynamics. The mutants lowered the RMSF values and maintained less fluctuation over time (Figure 3). A change in dynamics was observed in the mutants during simulations at a higher temperature, notably MUT143, which showed the biggest deviation (Figure 4). The mutants lowered the RMSF value in other parts of the protein and showed less fluctuation between residues compared to the wild type (Figure 5). Although the mutant residues did not return lower B-factor values at their position, Molecular Dynamics Simulation (MDS) showed that the mutations influenced the global flexibility profile of BaAXE, showing a lowered flexibility score and less fluctuation in the mutants. MDS is a useful tool for establishing a correlation between the protein structure and stability and is remarkably consistent with corresponding experimental data [9,46].

### 2.2. Mutant Residues

The properties of the mutant residues and their influence on the protein structure were computed in the WHAT IF server. The mutant and wild-type residues show similar B-factor values except that the side chain atoms of the mutant residues returned higher B-factor values compared to the wild-type. Contact analysis showed that the mutant A12D had additional intermolecular contacts and formed more hydrogen bonds compared to the wild type. A12D formed additional hydrogen bonding networks with SER10, PRO14, PRO44, and ASP45 while the wild-type residue A12 formed hydrogen bonding networks with PRO14 and PRO44. The number of interatomic contacts increased in the mutant residue A12D, most notably interatomic contact with ASP45 and a salt bridge with LYS16, which were not observed in the wild type (Table 2). The mutant Q143D showed no extra bonding nor interatomic contact but lost some of the hydrogen bonding networks with GLN140 observed in the wild-type residue GLU143 (Table 2). Furthermore, the mutant residue-aspartate (D) has a larger side chain compared to alanine (A) and would likely prevent the penetration of organic solvents into the protein core [30]. Increased stability of lipase in organic solvents has been linked to the introduction of amino acid residues that may be effective for preventing the organic solvent from entering the protein core [47]. The introduction of bulky aromatic residues to occupy solvent channels has been explored with success as a strategy to improve the stability of lipase in organic solvents [15]. The higher structural rigidity in thermophilic enzymes is thought to be accompanied by more inter- and intra-subunit interactions such as hydrophobic, hydrogen bonds, aromatic–aromatic, cation–aromatic, and disulphide bridges [48]. Amino acids with negatively or positively charged side chains can form salt bridges or hydrogen bonds and several other interactions that are not yet fully understood [49]. These interactions have been implicated in enhancing stability in the protein structure [35,50,51]. A study using high-resolution structures of 3150 polypeptide chains identified close pairs of carboxylates in acidic residues. These pairs of negatively charged residues were found to be tightly packed with low-B-factors, which supports the possible attractive interaction between D12 and D45 [52]. Furthermore, a novel π-π interaction was reportedly induced by introducing aromatic residues in a study to enhance the stability of lipase from *Geobacillus stearothermophilus* in methanol [15]. This emphasises the significance of intramolecular interactions in enhancing the stability of enzymes in organic solvents. Although aspartic acid and glutamic acid show similar hydrogen bonding attribute and are theoretically available to engage in carbonyl–carbonyl interaction since they both possess carbonyls in their side chains, glutamic acid show very little carbonyl stacking presumably owing to steric constraints [53]. Carbonyl–carbonyl interaction is more abundant in asparagine and aspartic acid compared to glutamine and glutamic acid. This might explain the absence of additional intermolecular interaction in the MUT143. Furthermore, substitutions to charged amino acids attracted more water to the enzyme surface and contributed to stronger attraction to the hydration shell [29]. Hence, an engineering surface charge can improve hydration and reduce flexibility.

The side chains of aspartic acid/glutamic acid residues can interact with the backbone amine groups/carbonyl groups forming the carbonyl–carbonyl interactions [54]. Carbonyl–carbonyl interactions (C=O⋯⋯C=O) is initiated when one of the lone pairs on the oxygen atom of one carbonyl group is delocalised over the antibonding orbital of a nearby carbonyl C=O bond [50]. Self-contacting residues are found in the N- or C-terminal ends of helices or loop regions and the majority if not all the self-contacting aspartate residue interact with at least one water molecule and/or with residues from other parts of the protein. The formation of carbonyl–carbonyl interaction results in optimum favourable interactions with low B-factor. Hence, the presence of such interactions could positively impact the protein stability [55]. Their presence in helix ends and loops indicates that these interactions can help fix the orientations of the helix ends or reduce the flexibility of the loop regions [56]. The structure of an endoglucanase (PDB: 1KS8) contains seven self-contacting residues, of which five of them are aspartic acid, one glutamine, and one glutamic acid [54]. All the self-contacting residues in this protein were interacting with water molecules and residues from other regions of the protein. The self-contacting residues formed at least one hydrogen bond with a water molecule and at least one with residues that are distant at the primary sequence level [54]. The proximity of two carbonyl oxygens with partial negative charges results in a repulsive effect between the like charges. However, this repulsive effect is offset by multiple favourable interactions occurring between the C=O groups and water molecule(s) and residues from other parts of the protein. The distance between the atom groups in the residues D12 and D45 averaged 3.307 ± 0.270, which are within distances for potential electrostatic contacts (Table 2). The crystallography data for 9049 carbon-substituted C=O groups show that 15% interact with other C=O groups where d(C-O) is less than 3.6 Å [57]. Mutation of self-contacting residues resulted in a loss of protein stability due to the disruption of stronger polar interactions in a relatively higher hydrophobic environment. Furthermore, proteins engineered to introduce these residues at appropriate positions resulted in stronger polar interaction networks leading to enhanced stability of the protein [57].

### 2.3. Enzyme Characterisics and Activity

The activity of the mutant enzymes was assayed in an assay with 4-nitrophenyl acetate. The mutants showed hydrolytic activity on 4-nitrophenyl acetate and showed deacetylation activity by the release of acetic acid when reacted with acetylated xylan and β-d-glucosepentaacetate as with the wild-type BaAXE [43]. When reacted with the substrate 4-nitrophenyl acetate, the mutants MUT12 and MUT12-143 reported Km values of 0.29 mM and 0.27 mM, respectively, while the wild type reported a Km value of 0.41 mM.

**Figure 6:**
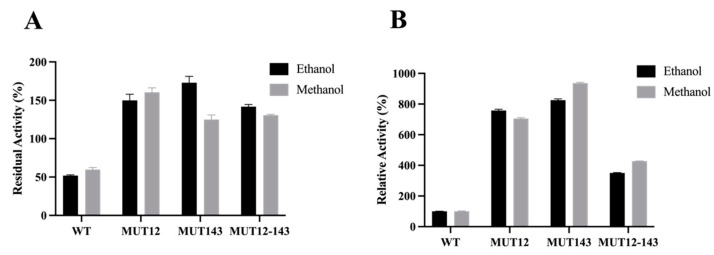
Performance in organic alcohol. Proteins were incubated in the presence of 20% hydrophilic organic solvents for 19 h. (**A**) The residual activity relative to enzymes without solvent incubation. (**B**) The activity of the mutant proteins relative to the wild-type protein after solvent incubation. Each bar represents triplicate data from each experiment and the error bar shows SEM (*n* = 3).

**Figure 7:**
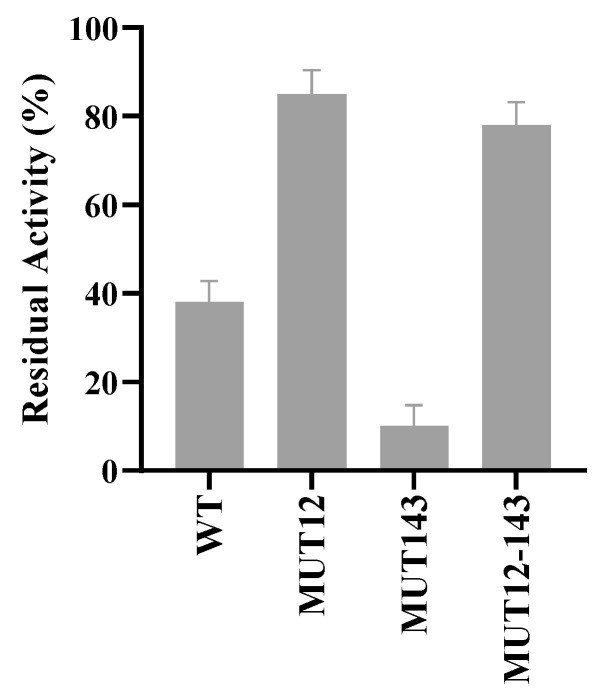
Stability in 40% methanol. The purified enzymes were incubated in 40% methanol for 19 h at 20 °C. Residual activity was measured in an assay with 4-nitrophenyl acetate. Each bar represents average data from replicate experiments. Error bar represents SEM where *n* = 4.

The performances of wild type and mutant variants were accessed in the presence of 20% and 40% organic solvents. The mutant enzymes showed higher relative and residual compared to the wild type in 20% ethanol and methanol (Figure 6). MUT12 and MUT143 showed over 700% higher activity in the presence of 20% hydrophilic organic solvent compared to the wild type (Figure 6). MUT12 and MUT12-143 showed 78% and 70% residual activity, respectively, and MUT12 retained less than 20% residual activity in 40% methanol (Figure 7). The mutants were more stable in hydrophilic organic solvents compared to the wild type. Interestingly, the mutants showed improved activity in hydrophilic solvents and MUT12 and MUT12-143 retained over 70% residual activity in 40% methanol. The loss of activity in MUT143 in 40% methanol could be explained by the lack of additional inter-molecular interactions and agree with the MDS profile where a steep change in RMSF was observed in MUT143 (Figure 4).

Amino acid mutation to charged residues had a positive effect on the activity and stability of BaAXE in hydrophilic organic solvents. This is interesting and remarkable as a previous attempt to enhance the activity and stability of a lipase in hydrophilic organic solvents was achieved by the simultaneous introduction of double mutants at the substrate-binding and hydrophilic solvent-affecting region [2]. Mutagenesis of residues located at the flexible regions away from the active sites has been reported capable of significantly improving enzyme catalysis. Enzyme catalysis is not only mediated by the catalytic pocket, but also the dynamic network associated with the active pocket and changes in structural conformation [14]. This explains the higher relative activity observed in the mutants compared to the wild type in 20% organic solvent (Figure 6). This work agrees to the postulation of improved hydration and reduced flexibility in enhancing the stability of enzymes in polar organic solvents [58]. In 20% organic solvent, the charged residues provided improved hydration resulting in enhanced activity and stability. However, in 40% organic solvent, the hydration shell is threatened by the increased strength of the organic solvent and would require the formation of intermolecular interactions to maintain stability (Figure 7). Hydrated shells formed by charged residues are stripped by hydrophilic organic solvents, hence the proposal for the removal of charged residues as a strategy for enhancing the stability of enzymes in organic solvents [59]. However, the surface charges in thermostable proteins are linked to the formation of salt bridges, and electrostatic interactions with charged amino acids have been implicated in maintaining the enzyme structure [60].

Based on the strong link between organic solvent resistance and thermostability [46], the improved stability observed in MUT12 and MUT12-143 in 40% methanol is likely to have been conferred by the introduction of a new interaction in the protein structure. Although it is not definite as to which interaction is responsible for the enhanced stability, the possible options would be the formation of hydrogen bonds, inter-residue interaction between the side-chain atoms and their main chain nitrogen or oxygen, and interaction with neighbouring residues.

## 3. Materials and Methods

### 3.1. Flexibility and Mutagenesis Analysis

The computationally developed structure of BaAXE as previously described was used for mutagenesis investigations [43]. B-factor value characterises the flexibility of a residue in a protein structure. The B-factor and local quality were estimated with ResQ server [61]. A high B-factor corresponds to greater flexibility and vice versa [36]. The modelled structure of BaAXE was divided into two regions—the substrate-binding region and the hydrophilic solvent affecting region [2]. Two residues—alanine (A12) and glutamine (Q143)—in the hydrophilic solvent-affecting region were selected for mutagenesis. The solvent-accessible surface area was calculated using the GetArea server (http://curie.utmb.edu/getarea.html, accessed on 11 April 2022) and the features of the mutated residues were computed with Protein Predict (https://predictprotein.org/, accessed on 11 April 2022).

### 3.2. Interatomic Contacts

The mutant residues were simulated into the computationally developed structure of BaAXE using the mutation prediction tool hosted in the WHAT IF server (https://swift.cmbi.umcn.nl/servers/html/index.html, accessed on 20 July 2022). The computationally developed mutant and wild-type structure was used to analyse the properties of mutant and wild-type residues, respectively. The interatomic contacts and salt bridges were analysed using the atomic contact tool while the hydrogen bonding network was analysed using the hydrogen (bonds) tool hosted in the WHAT IF server.

### 3.3. Molecular Dynamic Simulation

The structure of the mutant structure was developed using the computationally developed structure of BaAXE. The wild-type structure was mutated with the mutant residue using the mutation prediction tool hosted in the WHAT IF server. Molecular dynamic simulation was performed using Gromacs and the OPLS-AA/L all-atom force field. Due to the demand of the simulation on the remote system, remote runs were limited to 1 ns and simulations were performed at 300 K and 325 K. MDS was performed on the webGRO simulation (https://simlab.uams.edu/index.php, accessed on 30 August 2022) and simulations were extended to 20 ns at 325 K using this platform.

### 3.4. Generating Mutants

Site-directed mutagenesis was performed on the recombinant plasmid pDEST42:BaAXE through the substitution of oligonucleotides to achieve the desired mutants plasmids. Mutagenesis was performed with Q5^®^ Site-Directed Mutagenesis Kit following the manufacturer’s protocol (NEB, Hitchin, UK). Two mutant plasmids were constructed: (1) pDEST42:MUT12 having one mutant site—Ala12Asp, and (2) MUT12-143 having two mutant sites—Ala12Asp and Gln143Glu. The primers for mutagenesis are shown in (Table 3). The mutant genes were confirmed by restriction enzyme digest and sequencing (SourceBioscience, Cambridge, UK).

### 3.5. Transformation, Expression, and Purification of Recombinant Proteins

The recombinant plasmids confirmed through sequencing to harbour the mutant and wild-type genes were used to transform *Escherichia coli* BL21 (DE3) competent cells using chemical transformation with heat shock at 42 °C for 30 s. The transformed cells were grown for 16 h at 37 °C in LB media supplemented with 50 µg/mL carbenicillin. Cells at OD > 2 were diluted to achieve an OD of approximately 0.5. Diluted cells were grown to mid-log phase—OD 0.4–0.5 before protein expression was induced by adding 0.6 mM IPTG to the culture. The recombinant proteins were expressed at 30 °C for 4 h. Induced cells were harvested by centrifugation at 10,000× *g* for 10 min. Cell pellets were resuspended and incubated in a lysis buffer (50 mM Sodium phosphate, 150 mM NaCl, 0.01% triton X-100, 10 mM imidazole) for 30 min. The lysate was recovered by centrifugation at 4600 rpm for 15 min at 4 °C. Clarified lysate was passed through 0.22 µm filters and the filtrate was equilibrated 1:1 with an equilibration buffer (50 mM sodium phosphate, 150 mM NaCl, 10 mM imidazole pH 7.4). The equilibrated lysate was loaded onto gravity columns prefilled with cobalt resins (Cytiva, Sheffield, UK). His-tagged recombinant proteins were eluted with a gradient concentration of imidazole from 30 mM to 100 mM imidazole. The efficiency of the purification process was analysed with SDS-PAGE and Western blot. The eluents containing the purified proteins were dialysed using SnakeSkin dialysis tubing 10 K MWCO (Thermofischer, Leicester, UK) into a storage buffer (50 mM sodium phosphate, 100 mM NaCl) following the manufacturer’s protocol. After dialysis, the protein was concentrated using Amicon filters (Thermofischer, Leicester, UK) and quantified with Bradford assay.

### 3.6. Activity Assay

The activity of the recombinant enzymes was assayed on the synthetic substrate 4-nitrophenol acetate (4-NPA). The substrate was prepared to a concentration of 10 mM in DMSO. The assay reaction mixture contained 50 mM sodium phosphate pH 8 and 1 mM of the substrate. Enzyme reaction was initiated by adding 3.5–5 μg/mL of the purified enzyme, and an assay without the enzyme was used as a blank. The reaction mixture was incubated at 40 °C for 20 min with absorbance readings at 410 nm taken every 1 min using the BMG microplate reader (BMG Labtech, Aylesbury, UK). One unit of enzyme activity is defined as the amount of enzyme liberating 1 µmol of substrate per min under the reaction condition specified. Deacetylation activity of the mutant enzymes was accessed in an assay with acetylated xylan and β-d-glucosepentacetate as previously described [43]. Kinetic parameters Km and Kcat were determined as previously described [43].

### 3.7. Stability Assay in Organic Solvents

The organic solvent stability was determined by incubating the enzyme in 20% (*v*/*v*) ethanol and methanol, and 40% (*v*/*v*) methanol for 19 h at 20 °C. The residual activity of the enzymes after incubation was measured according to the method described above Section 3.4. The stability assay in 40% (*v*/*v*) hydrophilic organic solvent proceeded with just methanol because we observed from the 20% (*v*/*v*) assay that both methanol and ethanol had similar impact on the enzyme structure. The residual activity was expressed relative to the enzyme assay without alcohol incubation, and relative activity was expressed relative to the activity of the wild-type enzyme after alcohol incubation. Assay without an enzyme is used as a blank.

### 3.8. Stability in Acidic pH

To access the activity of the mutant enzymes in an acidic environment, an attribute lacking in the wild-type enzyme, the mutant enzymes were assayed according to Section 3.4 with sodium acetate buffer at pH 3 and 5. Assay without enzyme was used as a reaction blank. The activity of the mutant proteins was assessed relative to the wild-type enzyme.

## 4. Conclusions

Although the impact of substitutions to charged amino acids in enhancing enzyme stability is not fully understood, consistent hydration and reduced flexibility have been linked to the enhanced stability in hydrophilic organic solvents. Substitution to charged residues had a positive effect on residual activity in mild percentages of hydrophilic organic solvents while higher percentages of hydrophilic organic solvent would strip water from the protein surface leading to the loss of enzyme stability. It is likely that the introduced charged residue in position 12 (A12D) prevented the interaction of the cosolvent molecules with the enzyme by forming intermolecular interactions with residues on the enzyme surface and/or with water molecules. Hence, the enhanced stability in higher percentages of hydrophilic organic solvent. It would be informative to investigate the proposed interactions formed by the substitution A12D using experimentally determined structures with high resolution such as x-ray crystallography. Residues showing intra-residue interactions were found to be oriented with a consistent dihedral angle to engage interactions of significant energy [57]. Hence, the crystal structure of MUT12 would better explain the structural basis for the enhanced activity and stability in organic solvents. Furthermore, this rational approach can be explored in other residues (other regions/sites of the enzyme structure) to develop enhanced protein stability targeting different environments. For example, the mutants reported here showed no activity in the acidic pH range. However, the amide bridge interaction between the side-chain amide groups of asparagine and glutamine is reported to be three times stronger than hydrogen bonds and less influenced by the pH of the solution [49]. This strategy can aid in the rational design of stable and active acetyl xylan esterase in acidic pHs as most acetyl xylan esterases operate optimally in alkaline pH.

## Figures and Tables

**Table 1:** Mutant sites. The properties of the mutant residues were computed using the amino acid sequence of BaAXE. ^a^ Solvent accessible surface area reported in exposure ratio (%); ^b^ predicted normalised B-factor (normalised by Z-score transformation); ^c^ secondary structure.

Residue Number	Amino Acid		SASA (%) ^a^	nBF ^b^	SS ^c^
	Original	Mutant			
12	A	D	44.8	0.92	Loop
143	Q	E	71.6	0.99	Loop

**Table 2:** Interatomic contacts. The interatomic contacts were computed in the What IF server with the structure of the wild-type and mutant enzymes.

Residue	Atom (s)	Contact Residue	Contact Atom (s)	Ave. Distance
ASP12	Various	ASP45	Various	3.307 ± 0.270
ASP12	Various	SER10	Various	3.021 ± 0.503
ASP12	Various	LYS16	NZ	5.44 ± 0.07
ASP12	Various	PRO14	Various	3.26 ± 0.26
ASP12	Various	PRO44	Various	3.10 ± 0.33
ALA12	C	PRO14	CD	3.31
ALA12	N & CB	PRO44	O	3.02 ± 0.07
ALA12	N	SER10	C & OG	3.192 ± 0.20
GLU143	CB	GLN140	NE2	3.421
GLU143	C	ALA145	N	3.614
GLN143	Various	GLN140	Various	3.344 ± 0.43
GLN143	Various	ALA145	Various	3.614

**Table 3:** Primer sequences.

Mutant ID	Primer Sequence (5′–3′)
MUT12	FP: CAGAGTCCTGatACACCCGCAA RP: GACAACAAAGTGGTCATGTTTC
MUT143	FP: GCAGCTTCCAgAAAAAGCATC RP: GCGTAGCGACCATTAAAAG

The substituted oligonucleotides are written in lower case.

## Data Availability

Not applicable.
